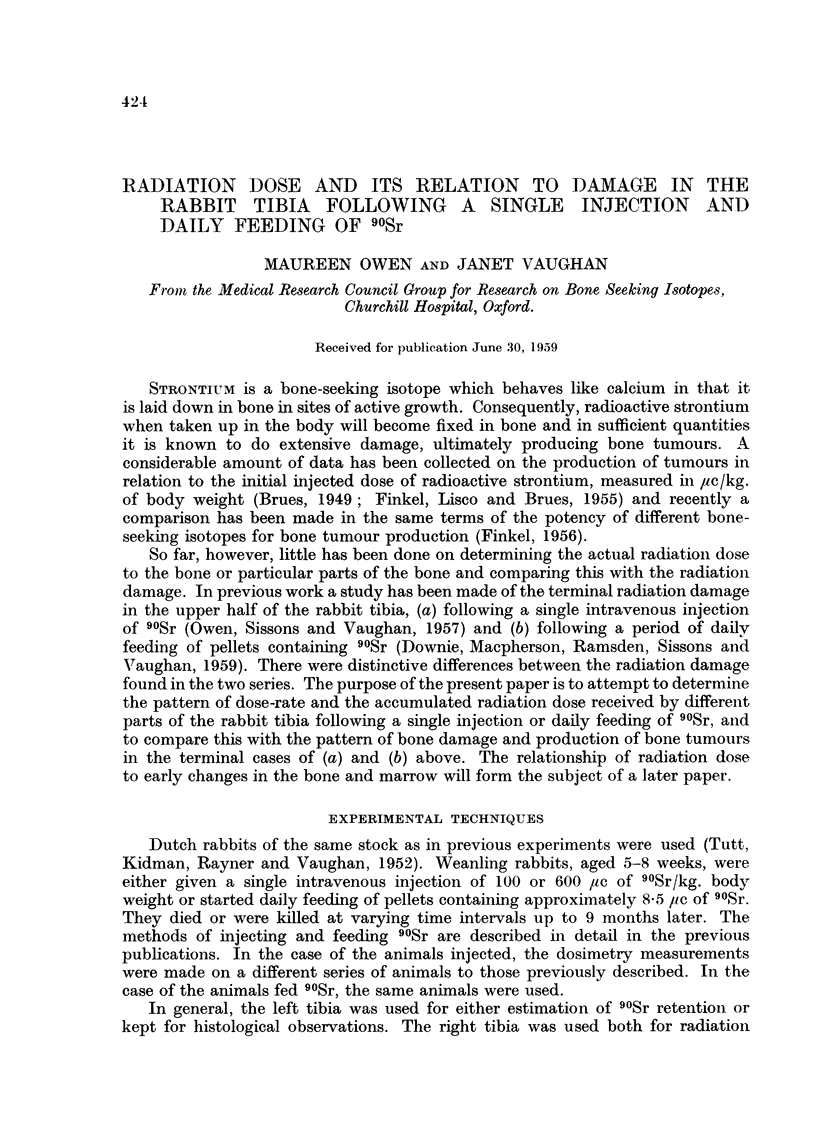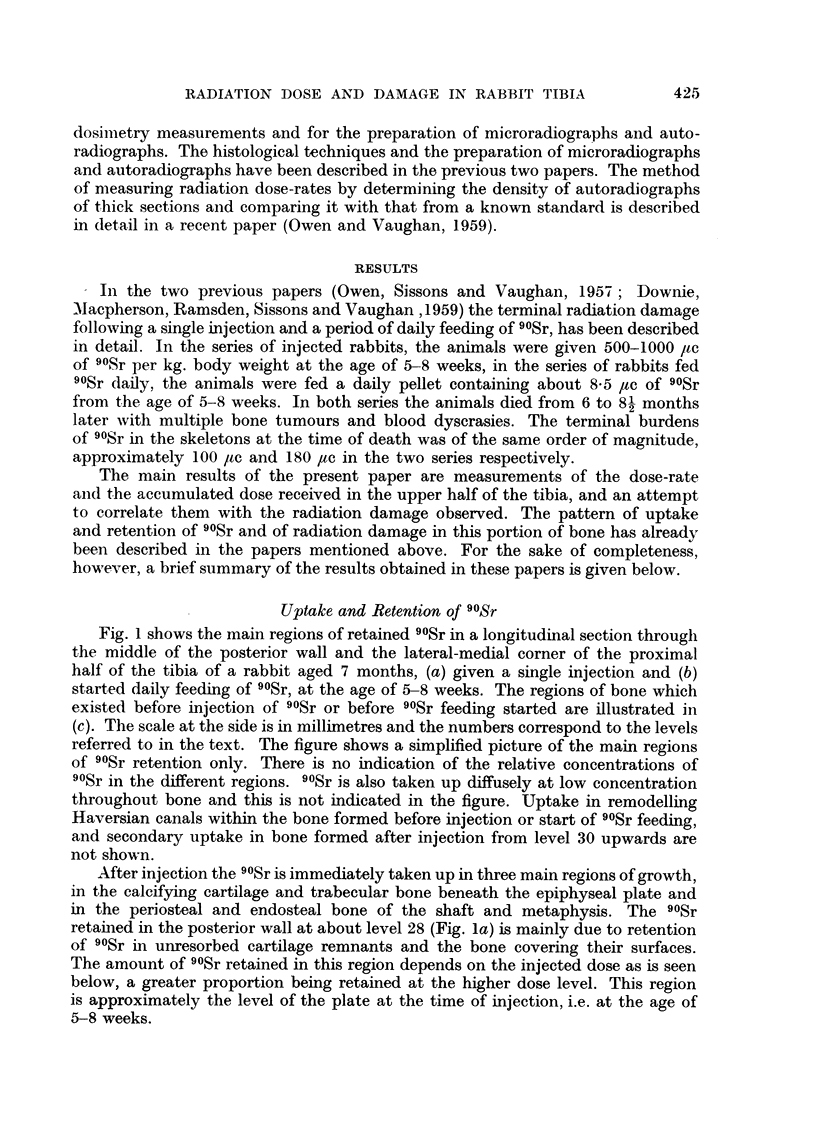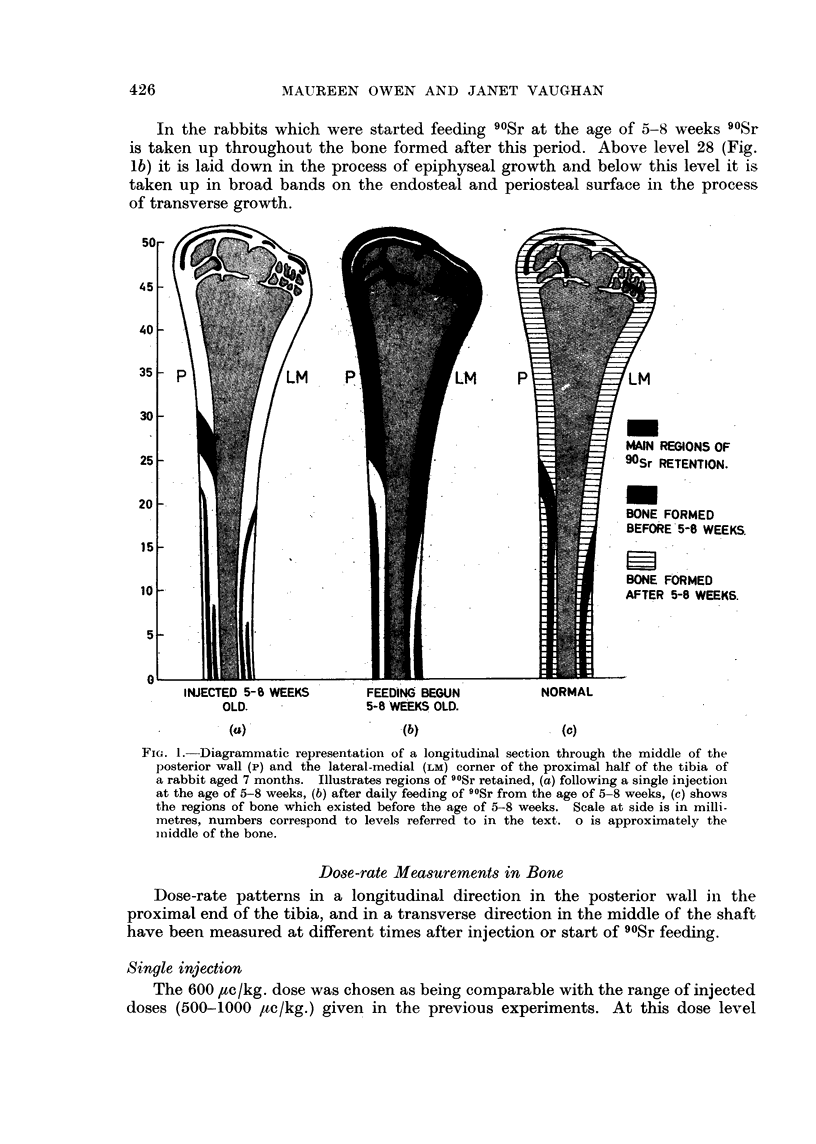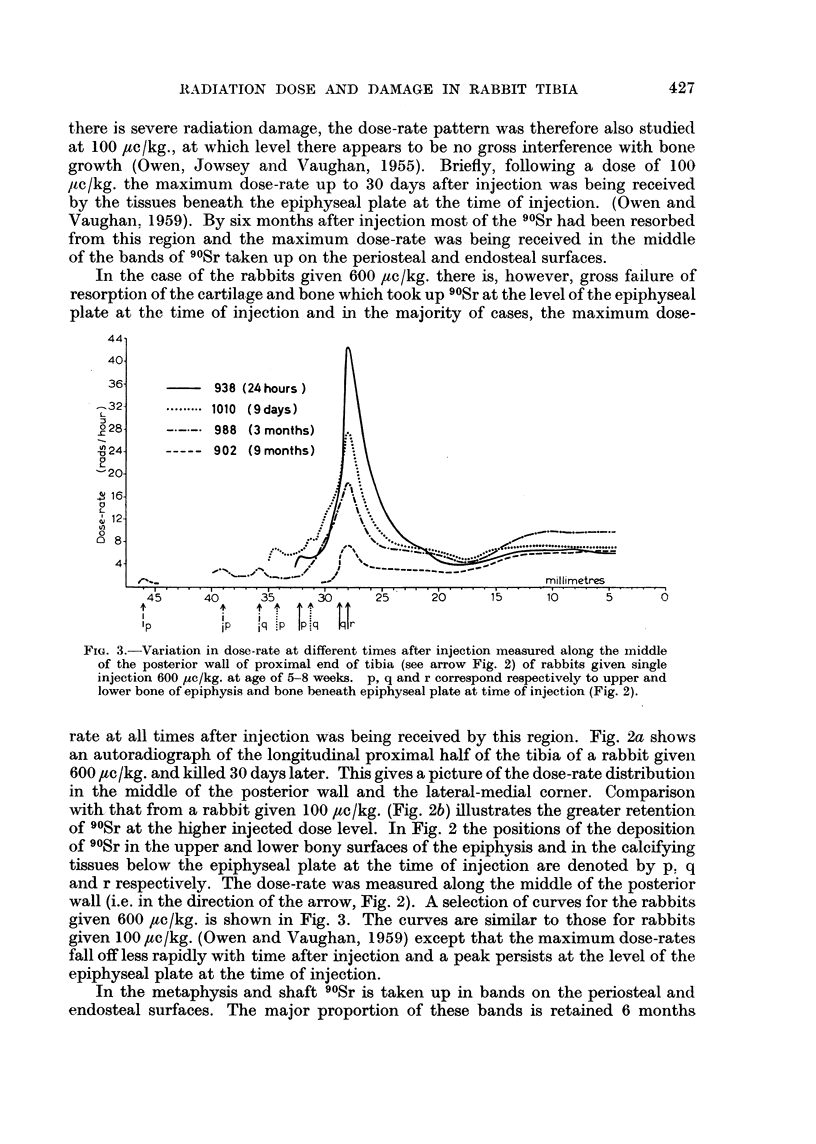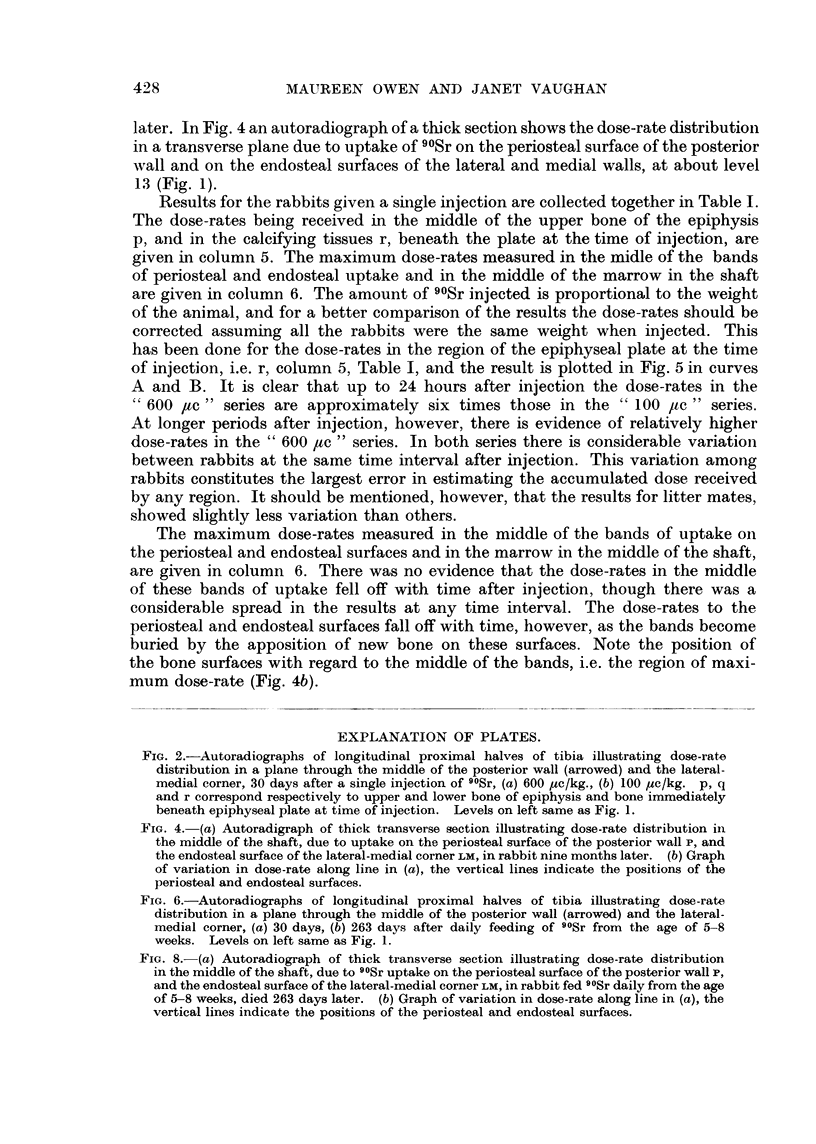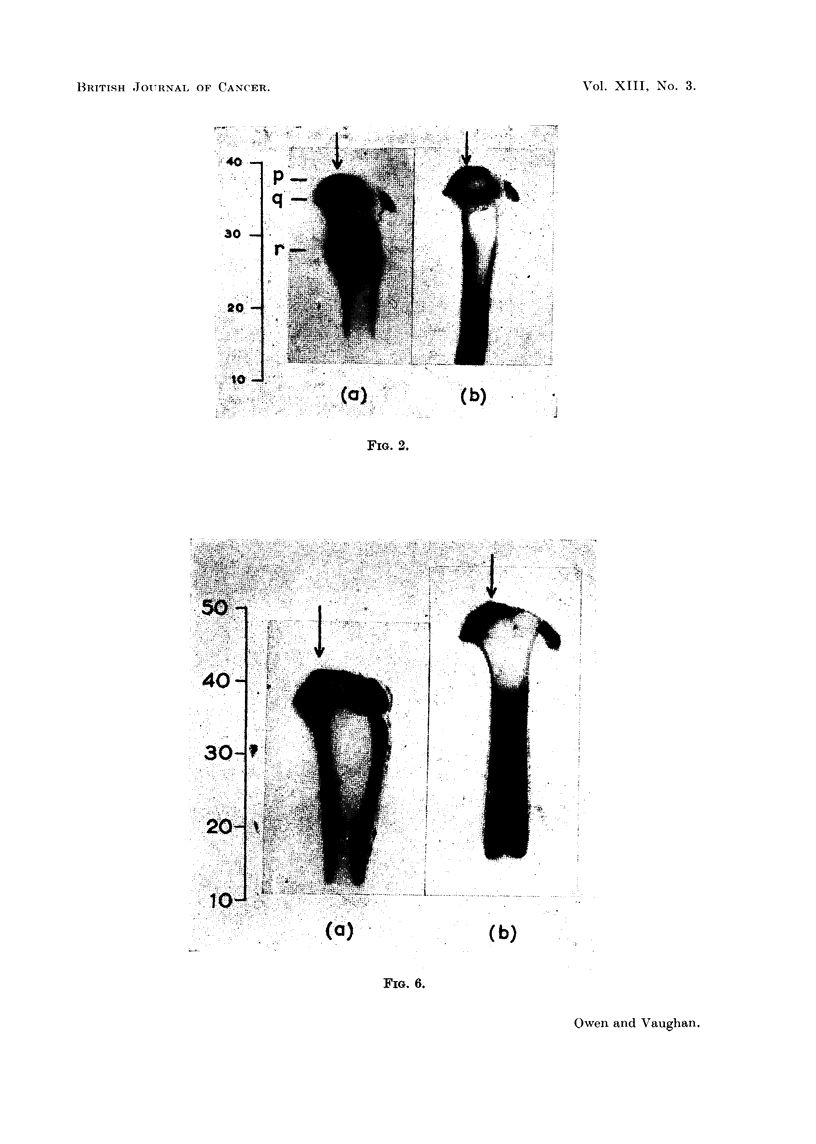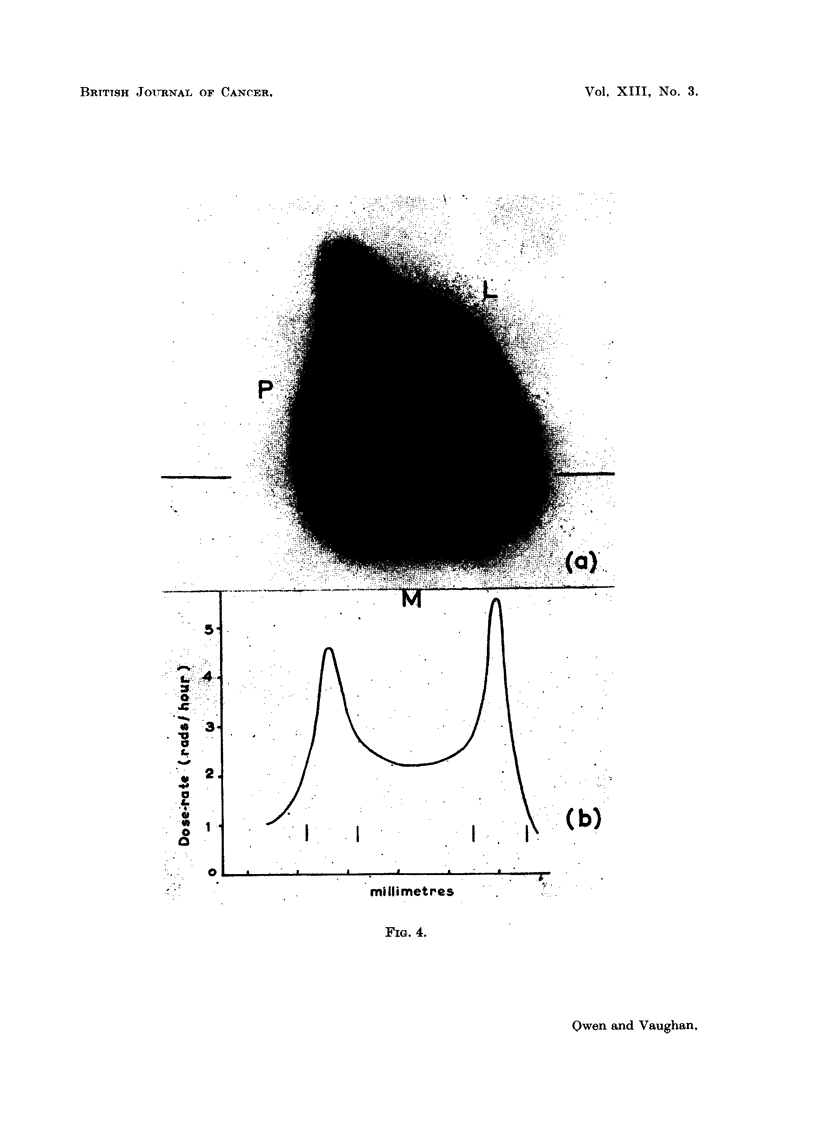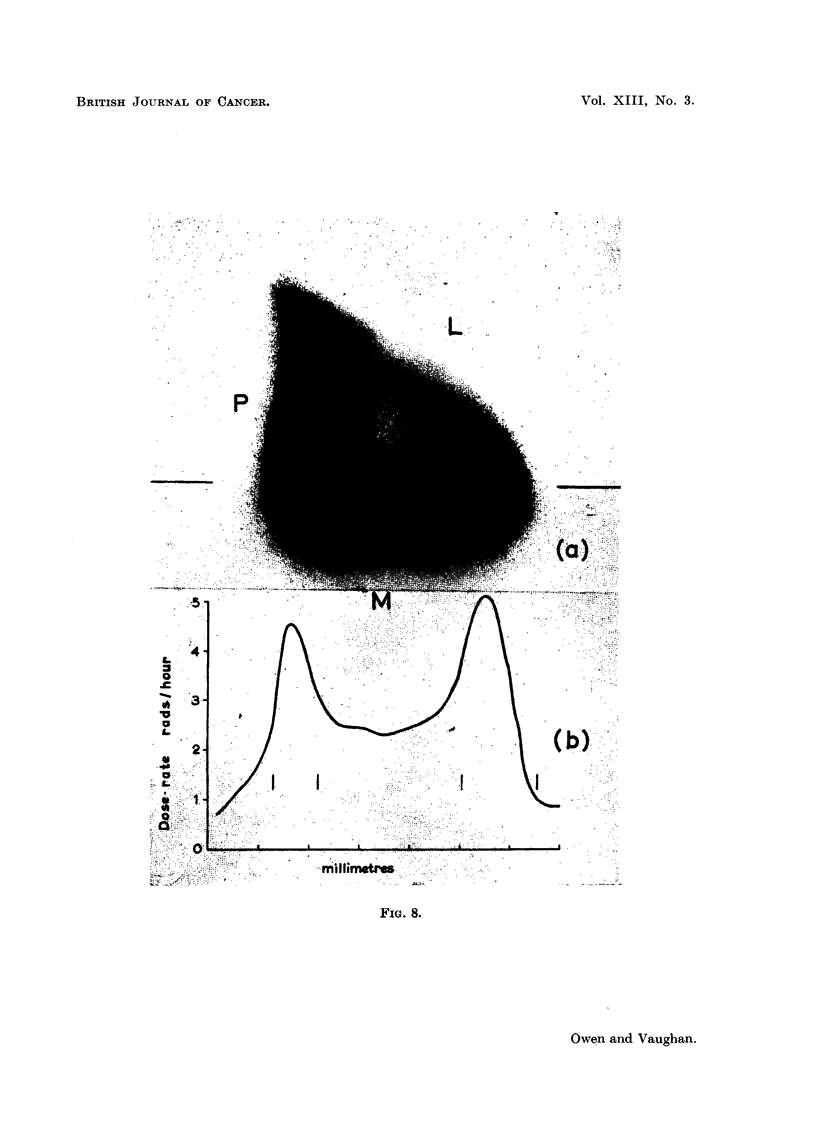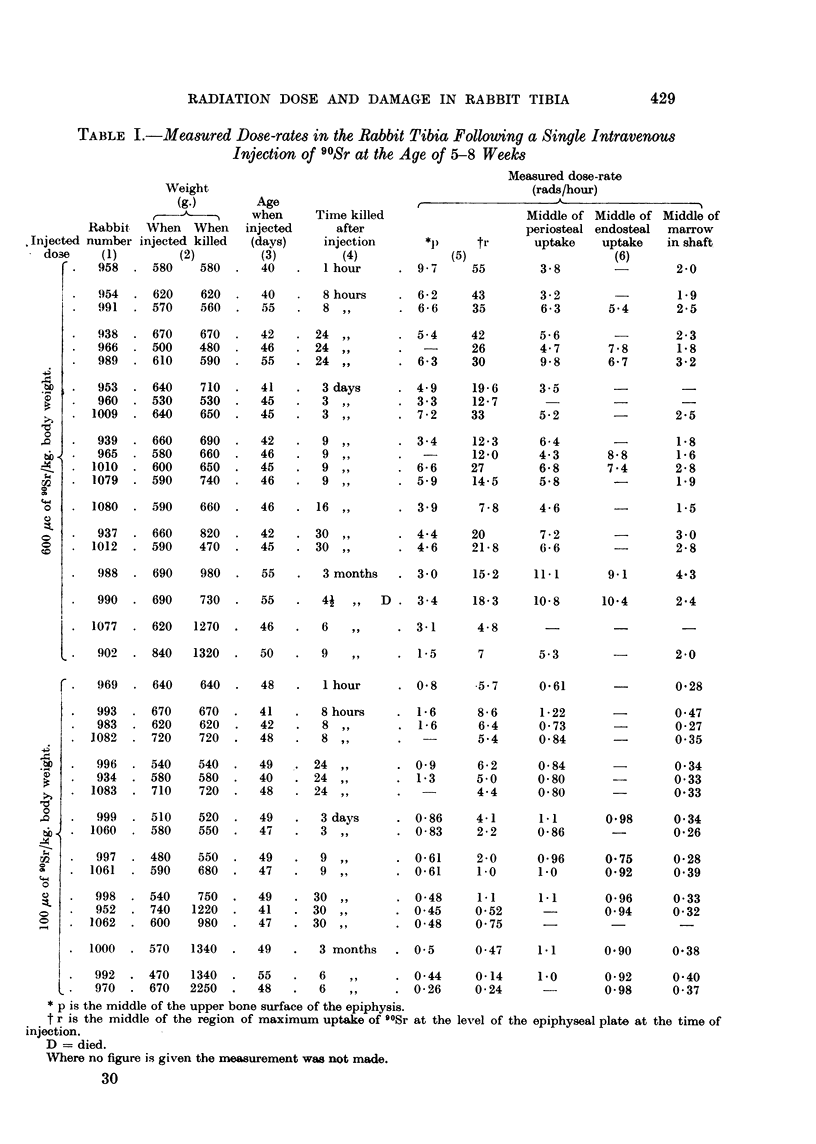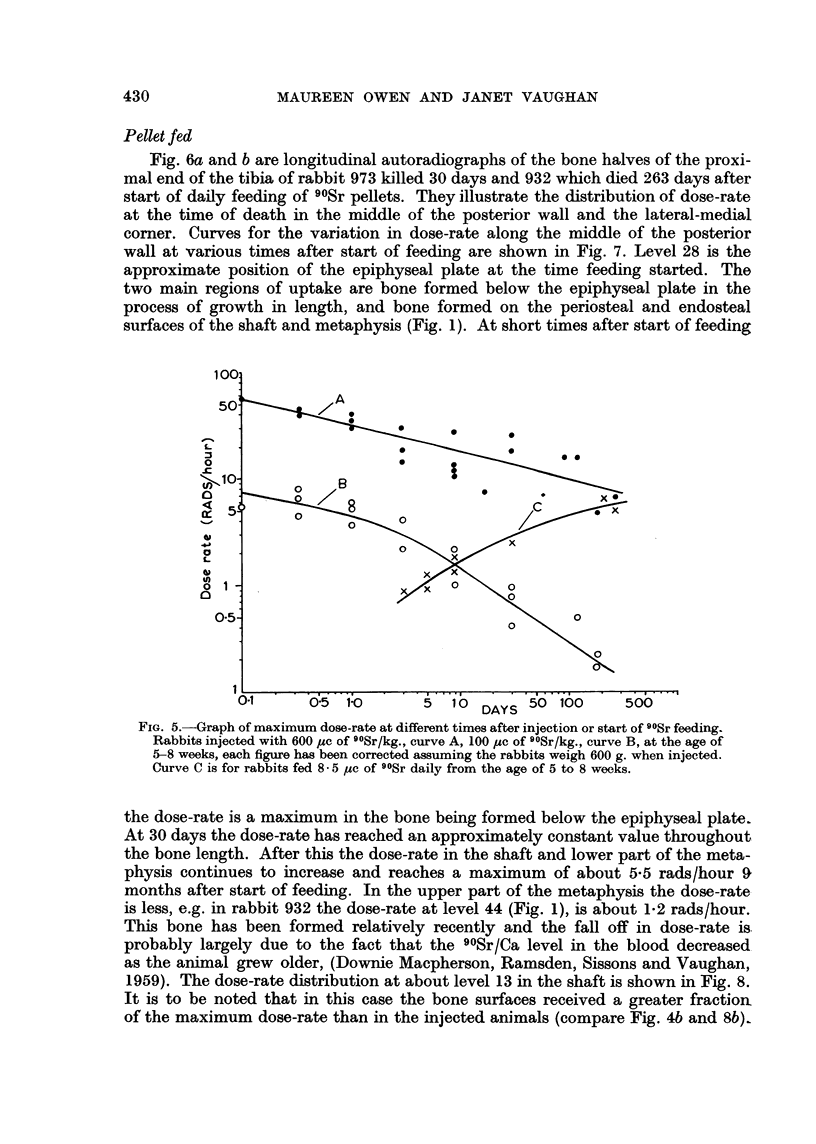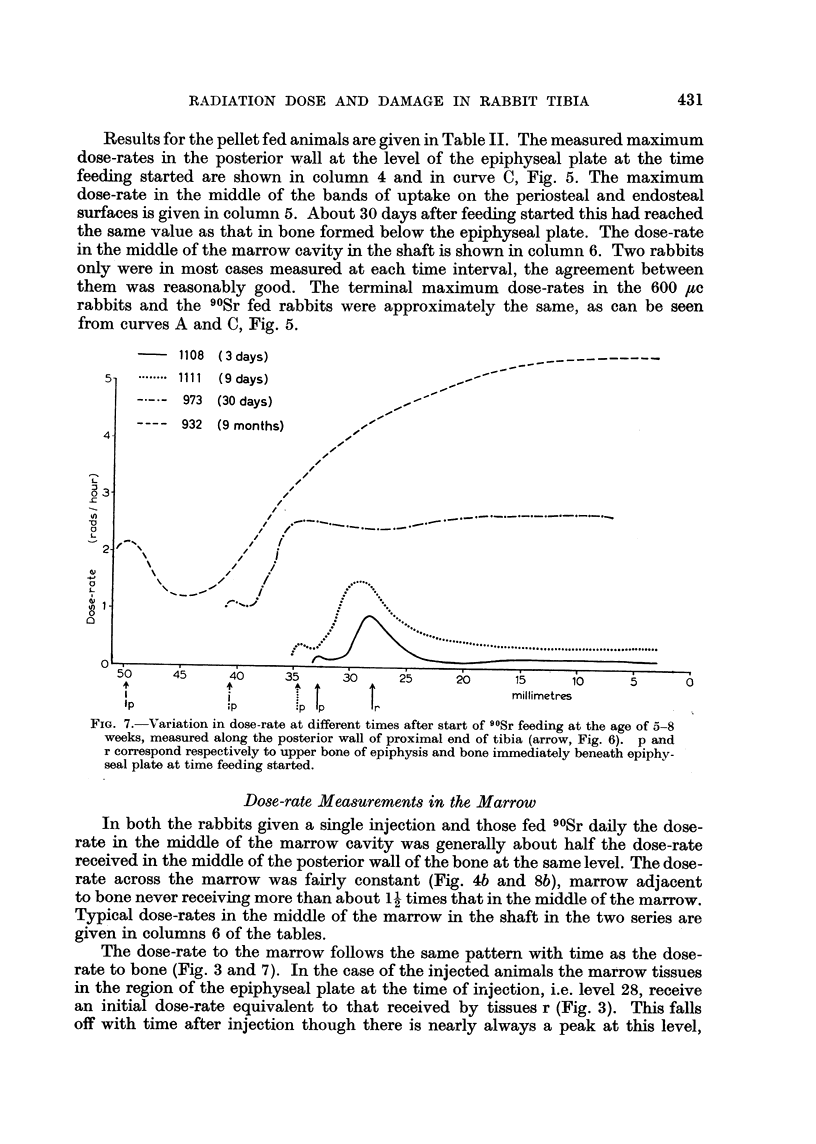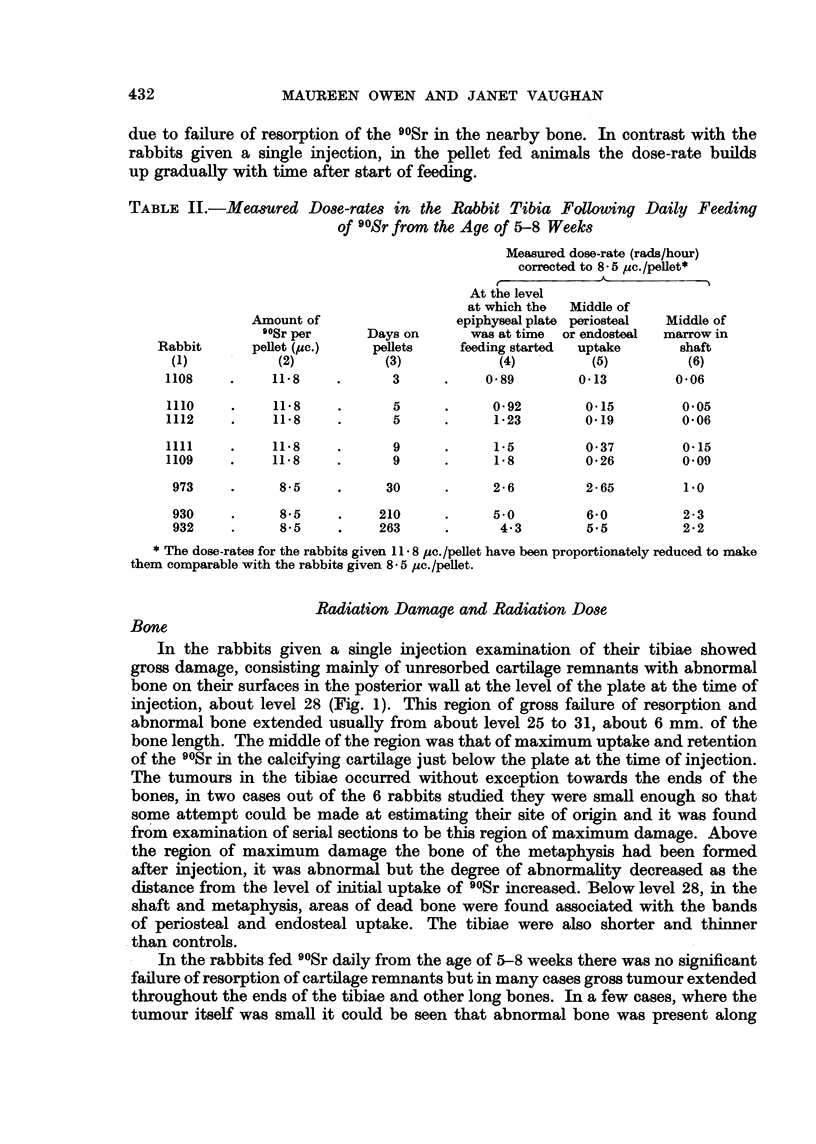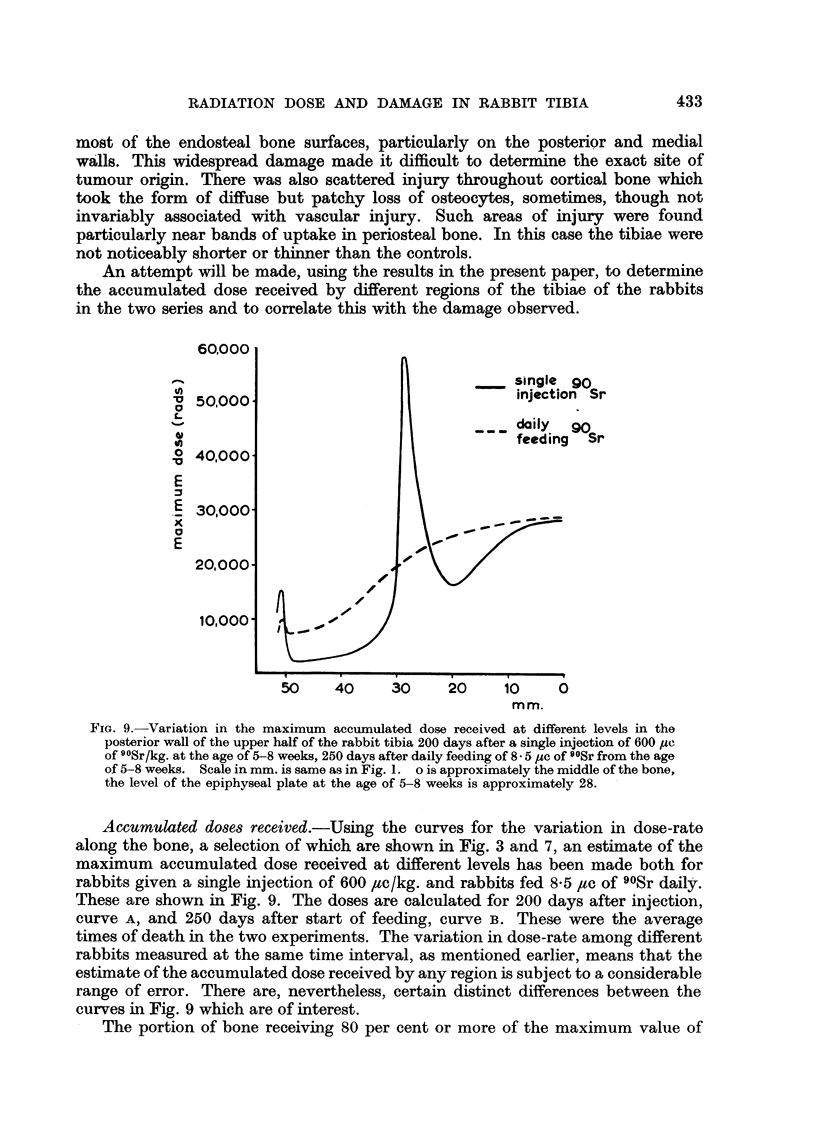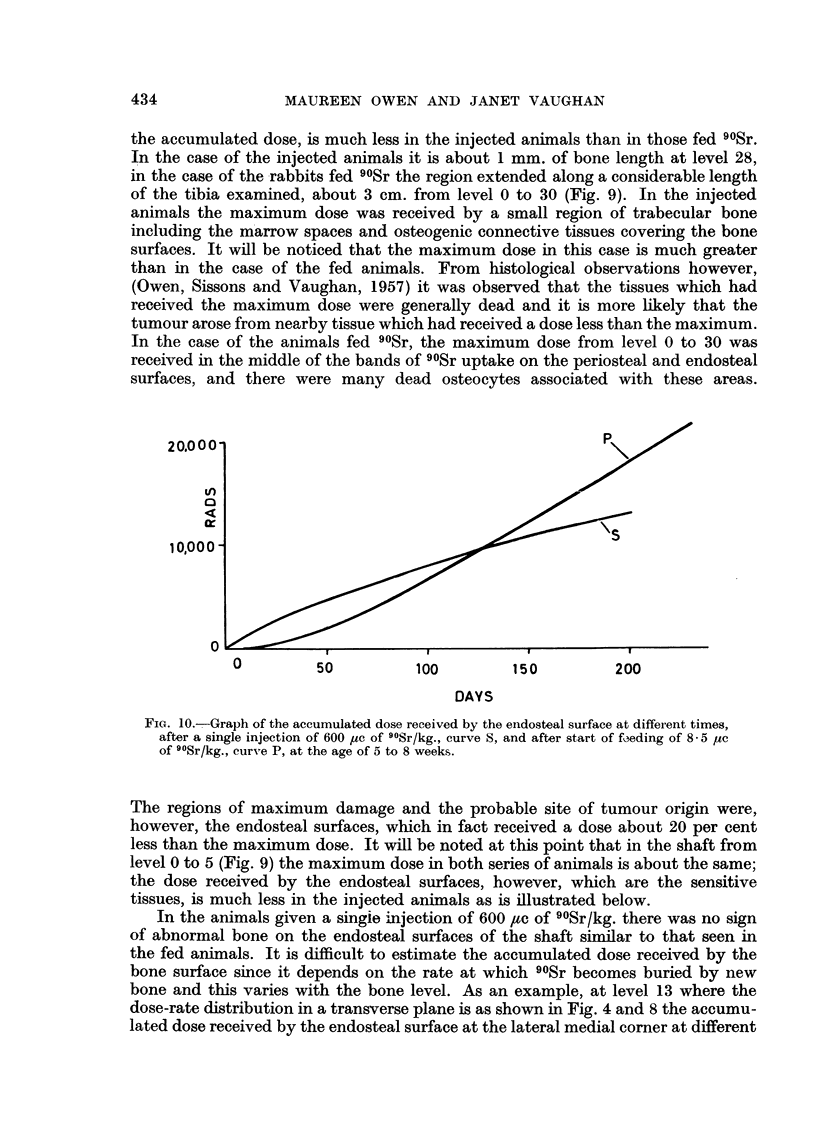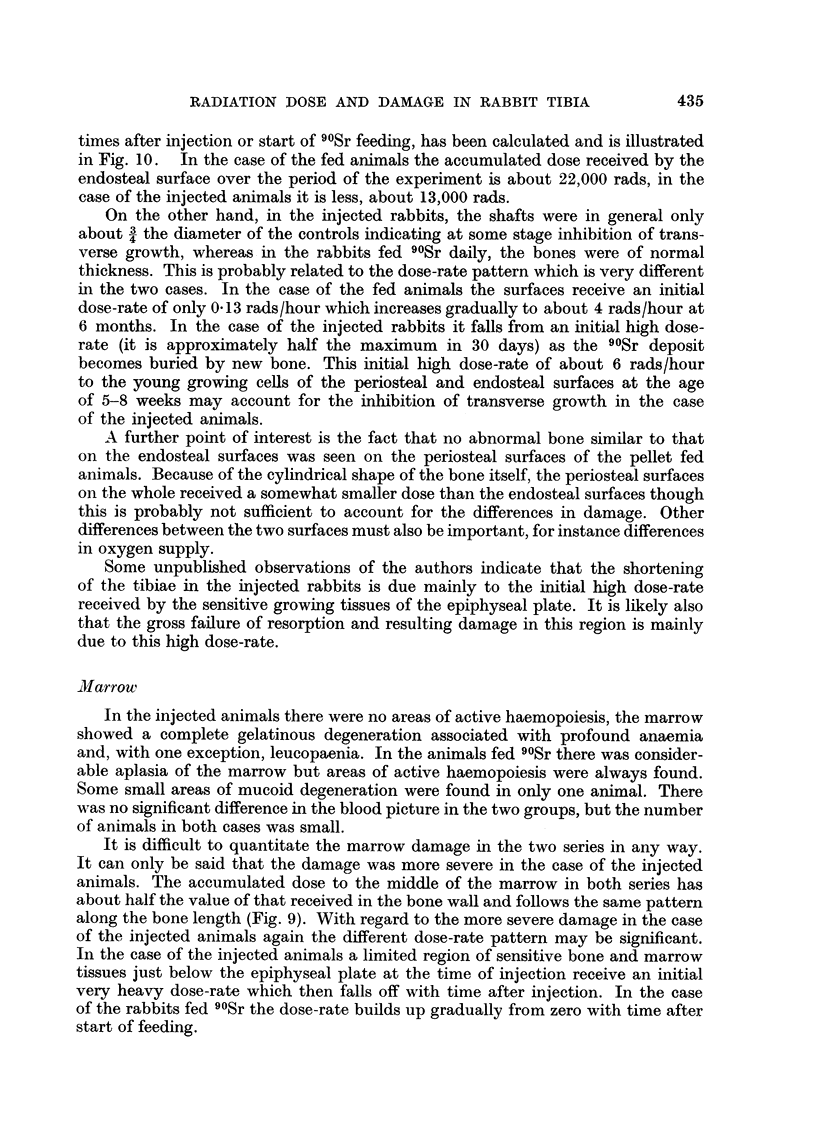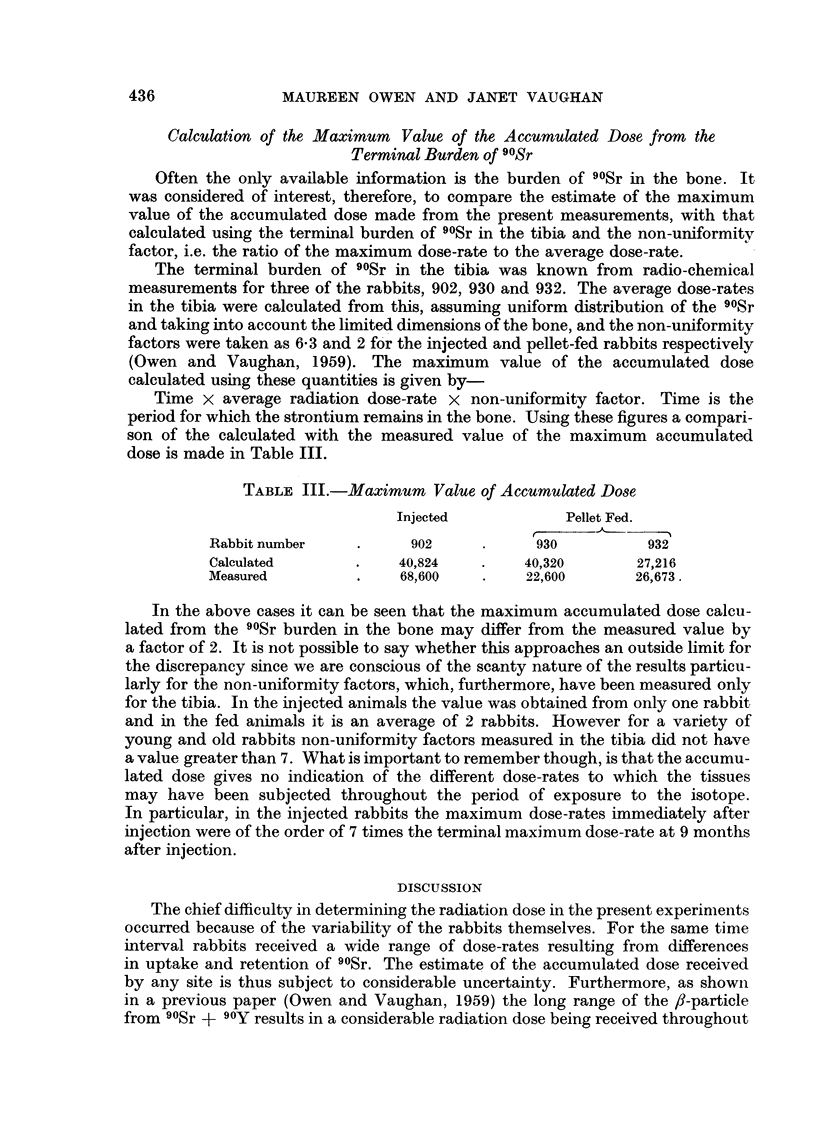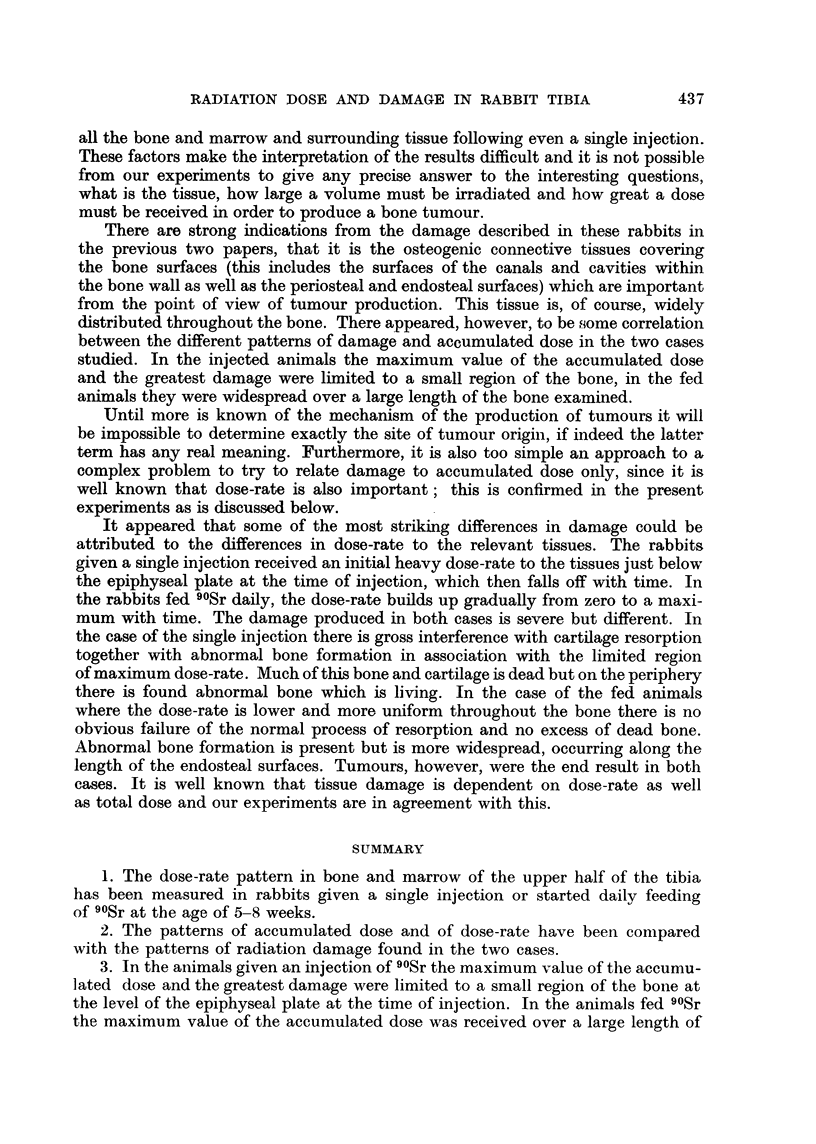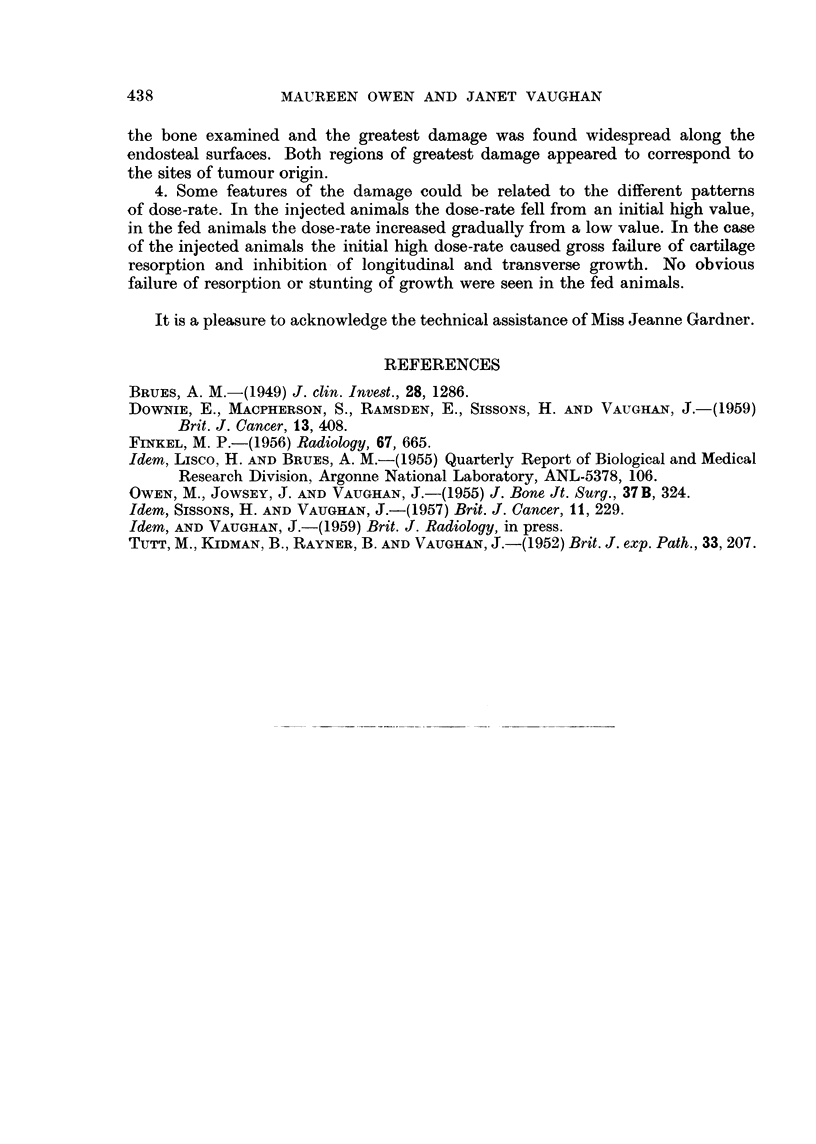# Radiation Dose and its Relation to Damage in the Rabbit Tibia Following a Single Injection and Daily Feeding of ^90^Sr

**DOI:** 10.1038/bjc.1959.48

**Published:** 1959-09

**Authors:** Maureen Owen, Janet Vaughan

## Abstract

**Images:**


### RADIATION DOSE AND ITS RELATION TO DAMAGE IN THE RABBIT TIBIA FOLLOWING A SINGLE INJECTION AND DAILY FEEDING OF $^{90}Sr$

MAUREEN OWEN AND JANET VAUGHAN

*From the Medical Research Council Group for Research on Bone Seeking Isotopes, Churchill Hospital, Oxford.*

Received for publication June 30, 1959

STRONTIUM is a bone-seeking isotope which behaves like calcium in that it is laid down in bone in sites of active growth. Consequently, radioactive strontium when taken up in the body will become fixed in bone and in sufficient quantities it is known to do extensive damage, ultimately producing bone tumours. A considerable amount of data has been collected on the production of tumours in relation to the initial injected dose of radioactive strontium, measured in $\mu$c/kg. of body weight (Brues, 1949; Finkel, Lisco and Brues, 1955) and recently a comparison has been made in the same terms of the potency of different bone-seeking isotopes for bone tumour production (Finkel, 1956).

So far, however, little has been done on determining the actual radiation dose to the bone or particular parts of the bone and comparing this with the radiation damage. In previous work a study has been made of the terminal radiation damage in the upper half of the rabbit tibia, (*a*) following a single intravenous injection of $^{90}Sr$ (Owen, Sissons and Vaughan, 1957) and (*b*) following a period of daily feeding of pellets containing $^{90}Sr$ (Downie, Macpherson, Ramsden, Sissons and Vaughan, 1959). There were distinctive differences between the radiation damage found in the two series. The purpose of the present paper is to attempt to determine the pattern of dose-rate and the accumulated radiation dose received by different parts of the rabbit tibia following a single injection or daily feeding of $^{90}Sr$, and to compare this with the pattern of bone damage and production of bone tumours in the terminal cases of (*a*) and (*b*) above. The relationship of radiation dose to early changes in the bone and marrow will form the subject of a later paper.

### EXPERIMENTAL TECHNIQUES

Dutch rabbits of the same stock as in previous experiments were used (Tutt, Kidman, Rayner and Vaughan, 1952). Weanling rabbits, aged 5–8 weeks, were either given a single intravenous injection of 100 or 600 $\mu$c of $^{90}Sr$/kg. body weight or started daily feeding of pellets containing approximately 8·5 $\mu$c of $^{90}Sr$. They died or were killed at varying time intervals up to 9 months later. The methods of injecting and feeding $^{90}Sr$ are described in detail in the previous publications. In the case of the animals injected, the dosimetry measurements were made on a different series of animals to those previously described. In the case of the animals fed $^{90}Sr$, the same animals were used.

In general, the left tibia was used for either estimation of $^{90}Sr$ retention or kept for histological observations. The right tibia was used both for radiation dosimetry measurements and for the preparation of microradiographs and autoradiographs. The histological techniques and the preparation of microradiographs and autoradiographs have been described in the previous two papers. The method of measuring radiation dose-rates by determining the density of autoradiographs of thick sections and comparing it with that from a known standard is described in detail in a recent paper (Owen and Vaughan, 1959).

### RESULTS

In the two previous papers (Owen, Sissons and Vaughan, 1957 ; Downie, Macpherson, Ramsden, Sissons and Vaughan ,1959) the terminal radiation damage following a single injection and a period of daily feeding of $^{90}$Sr, has been described in detail. In the series of injected rabbits, the animals were given 500–1000 $\mu$c of $^{90}$Sr per kg. body weight at the age of 5–8 weeks, in the series of rabbits fed $^{90}$Sr daily, the animals were fed a daily pellet containing about 8·5 $\mu$c of $^{90}$Sr from the age of 5–8 weeks. In both series the animals died from 6 to $8\frac{1}{2}$ months later with multiple bone tumours and blood dyscrasies. The terminal burdens of $^{90}$Sr in the skeletons at the time of death was of the same order of magnitude, approximately 100 $\mu$c and 180 $\mu$c in the two series respectively.

The main results of the present paper are measurements of the dose-rate and the accumulated dose received in the upper half of the tibia, and an attempt to correlate them with the radiation damage observed. The pattern of uptake and retention of $^{90}$Sr and of radiation damage in this portion of bone has already been described in the papers mentioned above. For the sake of completeness, however, a brief summary of the results obtained in these papers is given below.

### *Uptake and Retention of $^{90}Sr$*

Fig. 1 shows the main regions of retained $^{90}$Sr in a longitudinal section through the middle of the posterior wall and the lateral-medial corner of the proximal half of the tibia of a rabbit aged 7 months, (*a*) given a single injection and (*b*) started daily feeding of $^{90}$Sr, at the age of 5–8 weeks. The regions of bone which existed before injection of $^{90}$Sr or before $^{90}$Sr feeding started are illustrated in (*c*). The scale at the side is in millimetres and the numbers correspond to the levels referred to in the text. The figure shows a simplified picture of the main regions of $^{90}$Sr retention only. There is no indication of the relative concentrations of $^{90}$Sr in the different regions. $^{90}$Sr is also taken up diffusely at low concentration throughout bone and this is not indicated in the figure. Uptake in remodelling Haversian canals within the bone formed before injection or start of $^{90}$Sr feeding, and secondary uptake in bone formed after injection from level 30 upwards are not shown.

After injection the $^{90}$Sr is immediately taken up in three main regions of growth, in the calcifying cartilage and trabecular bone beneath the epiphyseal plate and in the periosteal and endosteal bone of the shaft and metaphysis. The $^{90}$Sr retained in the posterior wall at about level 28 (Fig. 1*a*) is mainly due to retention of $^{90}$Sr in unresorbed cartilage remnants and the bone covering their surfaces. The amount of $^{90}$Sr retained in this region depends on the injected dose as is seen below, a greater proportion being retained at the higher dose level. This region is approximately the level of the plate at the time of injection, i.e. at the age of 5–8 weeks.

In the rabbits which were started feeding $^{90}$Sr at the age of 5–8 weeks $^{90}$Sr is taken up throughout the bone formed after this period. Above level 28 (Fig. 1*b*) it is laid down in the process of epiphyseal growth and below this level it is taken up in broad bands on the endosteal and periosteal surface in the process of transverse growth.

FIG. 1.—Diagrammatic representation of a longitudinal section through the middle of the posterior wall (P) and the lateral-medial (LM) corner of the proximal half of the tibia of a rabbit aged 7 months. Illustrates regions of $^{90}$Sr retained, (*a*) following a single injection at the age of 5–8 weeks, (*b*) after daily feeding of $^{90}$Sr from the age of 5–8 weeks, (*c*) shows the regions of bone which existed before the age of 5–8 weeks. Scale at side is in millimetres, numbers correspond to levels referred to in the text. O is approximately the middle of the bone.

### *Dose-rate Measurements in Bone*

Dose-rate patterns in a longitudinal direction in the posterior wall in the proximal end of the tibia, and in a transverse direction in the middle of the shaft have been measured at different times after injection or start of $^{90}$Sr feeding.

### *Single injection*

The 600 $\mu$c/kg. dose was chosen as being comparable with the range of injected doses (500–1000 $\mu$c/kg.) given in the previous experiments. At this dose level there is severe radiation damage, the dose-rate pattern was therefore also studied at 100 $\mu$c/kg., at which level there appears to be no gross interference with bone growth (Owen, Jowsey and Vaughan, 1955). Briefly, following a dose of 100 $\mu$c/kg. the maximum dose-rate up to 30 days after injection was being received by the tissues beneath the epiphyseal plate at the time of injection. (Owen and Vaughan, 1959). By six months after injection most of the $^{90}$Sr had been resorbed from this region and the maximum dose-rate was being received in the middle of the bands of $^{90}$Sr taken up on the periosteal and endosteal surfaces.

In the case of the rabbits given 600 $\mu$c/kg. there is, however, gross failure of resorption of the cartilage and bone which took up $^{90}$Sr at the level of the epiphyseal plate at the time of injection and in the majority of cases, the maximum dose-

FIG. 3.—Variation in dose-rate at different times after injection measured along the middle of the posterior wall of proximal end of tibia (see arrow Fig. 2) of rabbits given single injection 600 $\mu$c/kg. at age of 5–8 weeks. p, q and r correspond respectively to upper and lower bone of epiphysis and bone beneath epiphyseal plate at time of injection (Fig. 2).

rate at all times after injection was being received by this region. Fig. 2*a* shows an autoradiograph of the longitudinal proximal half of the tibia of a rabbit given 600 $\mu$c/kg. and killed 30 days later. This gives a picture of the dose-rate distribution in the middle of the posterior wall and the lateral-medial corner. Comparison with that from a rabbit given 100 $\mu$c/kg. (Fig. 2*b*) illustrates the greater retention of $^{90}$Sr at the higher injected dose level. In Fig. 2 the positions of the deposition of $^{90}$Sr in the upper and lower bony surfaces of the epiphysis and in the calcifying tissues below the epiphyseal plate at the time of injection are denoted by p, q and r respectively. The dose-rate was measured along the middle of the posterior wall (i.e. in the direction of the arrow, Fig. 2). A selection of curves for the rabbits given 600 $\mu$c/kg. is shown in Fig. 3. The curves are similar to those for rabbits given 100 $\mu$c/kg. (Owen and Vaughan, 1959) except that the maximum dose-rates fall off less rapidly with time after injection and a peak persists at the level of the epiphyseal plate at the time of injection.

In the metaphysis and shaft $^{90}$Sr is taken up in bands on the periosteal and endosteal surfaces. The major proportion of these bands is retained 6 months later. In Fig. 4 an autoradiograph of a thick section shows the dose-rate distribution in a transverse plane due to uptake of $^{90}$Sr on the periosteal surface of the posterior wall and on the endosteal surfaces of the lateral and medial walls, at about level 13 (Fig. 1).

Results for the rabbits given a single injection are collected together in Table I. The dose-rates being received in the middle of the upper bone of the epiphysis p, and in the calcifying tissues r, beneath the plate at the time of injection, are given in column 5. The maximum dose-rates measured in the midle of the bands of periosteal and endosteal uptake and in the middle of the marrow in the shaft are given in column 6. The amount of $^{90}$Sr injected is proportional to the weight of the animal, and for a better comparison of the results the dose-rates should be corrected assuming all the rabbits were the same weight when injected. This has been done for the dose-rates in the region of the epiphyseal plate at the time of injection, i.e. r, column 5, Table I, and the result is plotted in Fig. 5 in curves A and B. It is clear that up to 24 hours after injection the dose-rates in the " 600 $\mu$c " series are approximately six times those in the " 100 $\mu$c " series. At longer periods after injection, however, there is evidence of relatively higher dose-rates in the " 600 $\mu$c " series. In both series there is considerable variation between rabbits at the same time interval after injection. This variation among rabbits constitutes the largest error in estimating the accumulated dose received by any region. It should be mentioned, however, that the results for litter mates, showed slightly less variation than others.

The maximum dose-rates measured in the middle of the bands of uptake on the periosteal and endosteal surfaces and in the marrow in the middle of the shaft, are given in column 6. There was no evidence that the dose-rates in the middle of these bands of uptake fell off with time after injection, though there was a considerable spread in the results at any time interval. The dose-rates to the periosteal and endosteal surfaces fall off with time, however, as the bands become buried by the apposition of new bone on these surfaces. Note the position of the bone surfaces with regard to the middle of the bands, i.e. the region of maximum dose-rate (Fig. 4*b*).

---

EXPLANATION OF PLATES.

Fig. 2.—Autoradiographs of longitudinal proximal halves of tibia illustrating dose-rate distribution in a plane through the middle of the posterior wall (arrowed) and the lateral-medial corner, 30 days after a single injection of $^{90}$Sr, (*a*) 600 $\mu$c/kg., (*b*) 100 $\mu$c/kg. p, q and r correspond respectively to upper and lower bone of epiphysis and bone immediately beneath epiphyseal plate at time of injection. Levels on left same as Fig. 1.

Fig. 4.—(*a*) Autoradigraph of thick transverse section illustrating dose-rate distribution in the middle of the shaft, due to uptake on the periosteal surface of the posterior wall P, and the endosteal surface of the lateral-medial corner LM, in rabbit nine months later. (*b*) Graph of variation in dose-rate along line in (*a*), the vertical lines indicate the positions of the periosteal and endosteal surfaces.

Fig. 6.—Autoradiographs of longitudinal proximal halves of tibia illustrating dose-rate distribution in a plane through the middle of the posterior wall (arrowed) and the lateral-medial corner, (*a*) 30 days, (*b*) 263 days after daily feeding of $^{90}$Sr from the age of 5–8 weeks. Levels on left same as Fig. 1.

Fig. 8.—(*a*) Autoradiograph of thick transverse section illustrating dose-rate distribution in the middle of the shaft, due to $^{90}$Sr uptake on the periosteal surface of the posterior wall P, and the endosteal surface of the lateral-medial corner LM, in rabbit fed $^{90}$Sr daily from the age of 5–8 weeks, died 263 days later. (*b*) Graph of variation in dose-rate along line in (*a*), the vertical lines indicate the positions of the periosteal and endosteal surfaces.

FIG. 2.

FIG. 6.

Owen and Vaughan.

FIG. 4.

FIG. 8.

TABLE I.—*Measured Dose-rates in the Rabbit Tibia Following a Single Intravenous Injection of $^{90}Sr$ at the Age of 5–8 Weeks*

| Injected dose | Rabbit number (1) | Weight (g.) When injected (2) | Weight (g.) When killed (2) | Age when injected (days) (3) | Time killed after injection (4) | Measured dose-rate (rads/hour) *p (5) | †r (5) | Middle of periosteal uptake | Middle of endosteal uptake (6) | Middle of marrow in shaft |
|---|---|---|---|---|---|---|---|---|---|---|
| 600 μc of $^{90}Sr$/kg. body weight. | 958 | 580 | 580 | 40 | 1 hour | 9·7 | 55 | 3·8 | — | 2·0 |
| | 954 | 620 | 620 | 40 | 8 hours | 6·2 | 43 | 3·2 | — | 1·9 |
| | 991 | 570 | 560 | 55 | 8 ,, | 6·6 | 35 | 6·3 | 5·4 | 2·5 |
| | 938 | 670 | 670 | 42 | 24 ,, | 5·4 | 42 | 5·6 | — | 2·3 |
| | 966 | 500 | 480 | 46 | 24 ,, | — | 26 | 4·7 | 7·8 | 1·8 |
| | 989 | 610 | 590 | 55 | 24 ,, | 6·3 | 30 | 9·8 | 6·7 | 3·2 |
| | 953 | 640 | 710 | 41 | 3 days | 4·9 | 19·6 | 3·5 | — | — |
| | 960 | 530 | 530 | 45 | 3 ,, | 3·3 | 12·7 | — | — | — |
| | 1009 | 640 | 650 | 45 | 3 ,, | 7·2 | 33 | 5·2 | — | 2·5 |
| | 939 | 660 | 690 | 42 | 9 ,, | 3·4 | 12·3 | 6·4 | — | 1·8 |
| | 965 | 580 | 660 | 46 | 9 ,, | — | 12·0 | 4·3 | 8·8 | 1·6 |
| | 1010 | 600 | 650 | 45 | 9 ,, | 6·6 | 27 | 6·8 | 7·4 | 2·8 |
| | 1079 | 590 | 740 | 46 | 9 ,, | 5·9 | 14·5 | 5·8 | — | 1·9 |
| | 1080 | 590 | 660 | 46 | 16 ,, | 3·9 | 7·8 | 4·6 | — | 1·5 |
| | 937 | 660 | 820 | 42 | 30 ,, | 4·4 | 20 | 7·2 | — | 3·0 |
| | 1012 | 590 | 470 | 45 | 30 ,, | 4·6 | 21·8 | 6·6 | — | 2·8 |
| | 988 | 690 | 980 | 55 | 3 months | 3·0 | 15·2 | 11·1 | 9·1 | 4·3 |
| | 990 | 690 | 730 | 55 | 4½ ,, D | 3·4 | 18·3 | 10·8 | 10·4 | 2·4 |
| | 1077 | 620 | 1270 | 46 | 6 ,, | 3·1 | 4·8 | — | — | — |
| | 902 | 840 | 1320 | 50 | 9 ,, | 1·5 | 7 | 5·3 | — | 2·0 |
| 100 μc of $^{90}Sr$/kg. body weight. | 969 | 640 | 640 | 48 | 1 hour | 0·8 | 5·7 | 0·61 | — | 0·28 |
| | 993 | 670 | 670 | 41 | 8 hours | 1·6 | 8·6 | 1·22 | — | 0·47 |
| | 983 | 620 | 620 | 42 | 8 ,, | 1·6 | 6·4 | 0·73 | — | 0·27 |
| | 1082 | 720 | 720 | 48 | 8 ,, | — | 5·4 | 0·84 | — | 0·35 |
| | 996 | 540 | 540 | 49 | 24 ,, | 0·9 | 6·2 | 0·84 | — | 0·34 |
| | 934 | 580 | 580 | 40 | 24 ,, | 1·3 | 5·0 | 0·80 | — | 0·33 |
| | 1083 | 710 | 720 | 48 | 24 ,, | — | 4·4 | 0·80 | — | 0·33 |
| | 999 | 510 | 520 | 49 | 3 days | 0·86 | 4·1 | 1·1 | 0·98 | 0·34 |
| | 1060 | 580 | 550 | 47 | 3 ,, | 0·83 | 2·2 | 0·86 | — | 0·26 |
| | 997 | 480 | 550 | 49 | 9 ,, | 0·61 | 2·0 | 0·96 | 0·75 | 0·28 |
| | 1061 | 590 | 680 | 47 | 9 ,, | 0·61 | 1·0 | 1·0 | 0·92 | 0·39 |
| | 998 | 540 | 750 | 49 | 30 ,, | 0·48 | 1·1 | 1·1 | 0·96 | 0·33 |
| | 952 | 740 | 1220 | 41 | 30 ,, | 0·45 | 0·52 | — | 0·94 | 0·32 |
| | 1062 | 600 | 980 | 47 | 30 ,, | 0·48 | 0·75 | — | — | — |
| | 1000 | 570 | 1340 | 49 | 3 months | 0·5 | 0·47 | 1·1 | 0·90 | 0·38 |
| | 992 | 470 | 1340 | 55 | 6 ,, | 0·44 | 0·14 | 1·0 | 0·92 | 0·40 |
| | 970 | 670 | 2250 | 48 | 6 ,, | 0·26 | 0·24 | — | 0·98 | 0·37 |

* p is the middle of the upper bone surface of the epiphysis.

† r is the middle of the region of maximum uptake of $^{90}Sr$ at the level of the epiphyseal plate at the time of injection.

D = died.

Where no figure is given the measurement was not made.

*Pellet fed*

Fig. 6*a* and *b* are longitudinal autoradiographs of the bone halves of the proximal end of the tibia of rabbit 973 killed 30 days and 932 which died 263 days after start of daily feeding of $^{90}$Sr pellets. They illustrate the distribution of dose-rate at the time of death in the middle of the posterior wall and the lateral-medial corner. Curves for the variation in dose-rate along the middle of the posterior wall at various times after start of feeding are shown in Fig. 7. Level 28 is the approximate position of the epiphyseal plate at the time feeding started. The two main regions of uptake are bone formed below the epiphyseal plate in the process of growth in length, and bone formed on the periosteal and endosteal surfaces of the shaft and metaphysis (Fig. 1). At short times after start of feeding

FIG. 5.—Graph of maximum dose-rate at different times after injection or start of $^{90}$Sr feeding. Rabbits injected with 600 $\mu$c of $^{90}$Sr/kg., curve A, 100 $\mu$c of $^{90}$Sr/kg., curve B, at the age of 5–8 weeks, each figure has been corrected assuming the rabbits weigh 600 g. when injected. Curve C is for rabbits fed 8·5 $\mu$c of $^{90}$Sr daily from the age of 5 to 8 weeks.

the dose-rate is a maximum in the bone being formed below the epiphyseal plate. At 30 days the dose-rate has reached an approximately constant value throughout the bone length. After this the dose-rate in the shaft and lower part of the metaphysis continues to increase and reaches a maximum of about 5·5 rads/hour 9 months after start of feeding. In the upper part of the metaphysis the dose-rate is less, e.g. in rabbit 932 the dose-rate at level 44 (Fig. 1), is about 1·2 rads/hour. This bone has been formed relatively recently and the fall off in dose-rate is probably largely due to the fact that the $^{90}$Sr/Ca level in the blood decreased as the animal grew older, (Downie Macpherson, Ramsden, Sissons and Vaughan, 1959). The dose-rate distribution at about level 13 in the shaft is shown in Fig. 8. It is to be noted that in this case the bone surfaces received a greater fraction of the maximum dose-rate than in the injected animals (compare Fig. 4*b* and 8*b*).

Results for the pellet fed animals are given in Table II. The measured maximum dose-rates in the posterior wall at the level of the epiphyseal plate at the time feeding started are shown in column 4 and in curve C, Fig. 5. The maximum dose-rate in the middle of the bands of uptake on the periosteal and endosteal surfaces is given in column 5. About 30 days after feeding started this had reached the same value as that in bone formed below the epiphyseal plate. The dose-rate in the middle of the marrow cavity in the shaft is shown in column 6. Two rabbits only were in most cases measured at each time interval, the agreement between them was reasonably good. The terminal maximum dose-rates in the 600 $\mu c$ rabbits and the $^{90}Sr$ fed rabbits were approximately the same, as can be seen from curves A and C, Fig. 5.

FIG. 7.—Variation in dose-rate at different times after start of $^{90}Sr$ feeding at the age of 5–8 weeks, measured along the posterior wall of proximal end of tibia (arrow, Fig. 6). p and r correspond respectively to upper bone of epiphysis and bone immediately beneath epiphyseal plate at time feeding started.

### *Dose-rate Measurements in the Marrow*

In both the rabbits given a single injection and those fed $^{90}Sr$ daily the dose-rate in the middle of the marrow cavity was generally about half the dose-rate received in the middle of the posterior wall of the bone at the same level. The dose-rate across the marrow was fairly constant (Fig. 4*b* and 8*b*), marrow adjacent to bone never receiving more than about $1\frac{1}{2}$ times that in the middle of the marrow. Typical dose-rates in the middle of the marrow in the shaft in the two series are given in columns 6 of the tables.

The dose-rate to the marrow follows the same pattern with time as the dose-rate to bone (Fig. 3 and 7). In the case of the injected animals the marrow tissues in the region of the epiphyseal plate at the time of injection, i.e. level 28, receive an initial dose-rate equivalent to that received by tissues r (Fig. 3). This falls off with time after injection though there is nearly always a peak at this level, due to failure of resorption of the $^{90}$Sr in the nearby bone. In contrast with the rabbits given a single injection, in the pellet fed animals the dose-rate builds up gradually with time after start of feeding.

TABLE II.—*Measured Dose-rates in the Rabbit Tibia Following Daily Feeding of $^{90}$Sr from the Age of 5–8 Weeks*

| Rabbit (1) | Amount of $^{90}$Sr per pellet (μc.) (2) | Days on pellets (3) | Measured dose-rate (rads/hour) corrected to 8·5 μc./pellet* At the level at which the epiphyseal plate was at time feeding started (4) | Middle of periosteal or endosteal uptake (5) | Middle of marrow in shaft (6) |
|---|---|---|---|---|---|
| 1108 | 11·8 | 3 | 0·89 | 0·13 | 0·06 |
| 1110 | 11·8 | 5 | 0·92 | 0·15 | 0·05 |
| 1112 | 11·8 | 5 | 1·23 | 0·19 | 0·06 |
| 1111 | 11·8 | 9 | 1·5 | 0·37 | 0·15 |
| 1109 | 11·8 | 9 | 1·8 | 0·26 | 0·09 |
| 973 | 8·5 | 30 | 2·6 | 2·65 | 1·0 |
| 930 | 8·5 | 210 | 5·0 | 6·0 | 2·3 |
| 932 | 8·5 | 263 | 4·3 | 5·5 | 2·2 |

* The dose-rates for the rabbits given 11·8 μc./pellet have been proportionately reduced to make them comparable with the rabbits given 8·5 μc./pellet.

### *Radiation Damage and Radiation Dose*

### *Bone*

In the rabbits given a single injection examination of their tibiae showed gross damage, consisting mainly of unresorbed cartilage remnants with abnormal bone on their surfaces in the posterior wall at the level of the plate at the time of injection, about level 28 (Fig. 1). This region of gross failure of resorption and abnormal bone extended usually from about level 25 to 31, about 6 mm. of the bone length. The middle of the region was that of maximum uptake and retention of the $^{90}$Sr in the calcifying cartilage just below the plate at the time of injection. The tumours in the tibiae occurred without exception towards the ends of the bones, in two cases out of the 6 rabbits studied they were small enough so that some attempt could be made at estimating their site of origin and it was found from examination of serial sections to be this region of maximum damage. Above the region of maximum damage the bone of the metaphysis had been formed after injection, it was abnormal but the degree of abnormality decreased as the distance from the level of initial uptake of $^{90}$Sr increased. Below level 28, in the shaft and metaphysis, areas of dead bone were found associated with the bands of periosteal and endosteal uptake. The tibiae were also shorter and thinner than controls.

In the rabbits fed $^{90}$Sr daily from the age of 5–8 weeks there was no significant failure of resorption of cartilage remnants but in many cases gross tumour extended throughout the ends of the tibiae and other long bones. In a few cases, where the tumour itself was small it could be seen that abnormal bone was present along most of the endosteal bone surfaces, particularly on the posterior and medial walls. This widespread damage made it difficult to determine the exact site of tumour origin. There was also scattered injury throughout cortical bone which took the form of diffuse but patchy loss of osteocytes, sometimes, though not invariably associated with vascular injury. Such areas of injury were found particularly near bands of uptake in periosteal bone. In this case the tibiae were not noticeably shorter or thinner than the controls.

An attempt will be made, using the results in the present paper, to determine the accumulated dose received by different regions of the tibiae of the rabbits in the two series and to correlate this with the damage observed.

FIG. 9.—Variation in the maximum accumulated dose received at different levels in the posterior wall of the upper half of the rabbit tibia 200 days after a single injection of 600 $\mu c$ of $^{90}Sr$/kg. at the age of 5–8 weeks, 250 days after daily feeding of 8·5 $\mu c$ of $^{90}Sr$ from the age of 5–8 weeks. Scale in mm. is same as in Fig. 1. O is approximately the middle of the bone, the level of the epiphyseal plate at the age of 5–8 weeks is approximately 28.

*Accumulated doses received.*—Using the curves for the variation in dose-rate along the bone, a selection of which are shown in Fig. 3 and 7, an estimate of the maximum accumulated dose received at different levels has been made both for rabbits given a single injection of 600 $\mu c$/kg. and rabbits fed 8·5 $\mu c$ of $^{90}Sr$ daily. These are shown in Fig. 9. The doses are calculated for 200 days after injection, curve A, and 250 days after start of feeding, curve B. These were the average times of death in the two experiments. The variation in dose-rate among different rabbits measured at the same time interval, as mentioned earlier, means that the estimate of the accumulated dose received by any region is subject to a considerable range of error. There are, nevertheless, certain distinct differences between the curves in Fig. 9 which are of interest.

The portion of bone receiving 80 per cent or more of the maximum value of the accumulated dose, is much less in the injected animals than in those fed $^{90}Sr$. In the case of the injected animals it is about 1 mm. of bone length at level 28, in the case of the rabbits fed $^{90}Sr$ the region extended along a considerable length of the tibia examined, about 3 cm. from level 0 to 30 (Fig. 9). In the injected animals the maximum dose was received by a small region of trabecular bone including the marrow spaces and osteogenic connective tissues covering the bone surfaces. It will be noticed that the maximum dose in this case is much greater than in the case of the fed animals. From histological observations however, (Owen, Sissons and Vaughan, 1957) it was observed that the tissues which had received the maximum dose were generally dead and it is more likely that the tumour arose from nearby tissue which had received a dose less than the maximum. In the case of the animals fed $^{90}Sr$, the maximum dose from level 0 to 30 was received in the middle of the bands of $^{90}Sr$ uptake on the periosteal and endosteal surfaces, and there were many dead osteocytes associated with these areas.

FIG. 10.—Graph of the accumulated dose received by the endosteal surface at different times, after a single injection of 600 $\mu c$ of $^{90}Sr$/kg., curve S, and after start of feeding of 8·5 $\mu c$ of $^{90}Sr$/kg., curve P, at the age of 5 to 8 weeks.

The regions of maximum damage and the probable site of tumour origin were, however, the endosteal surfaces, which in fact received a dose about 20 per cent less than the maximum dose. It will be noted at this point that in the shaft from level 0 to 5 (Fig. 9) the maximum dose in both series of animals is about the same; the dose received by the endosteal surfaces, however, which are the sensitive tissues, is much less in the injected animals as is illustrated below.

In the animals given a single injection of 600 $\mu c$ of $^{90}Sr$/kg. there was no sign of abnormal bone on the endosteal surfaces of the shaft similar to that seen in the fed animals. It is difficult to estimate the accumulated dose received by the bone surface since it depends on the rate at which $^{90}Sr$ becomes buried by new bone and this varies with the bone level. As an example, at level 13 where the dose-rate distribution in a transverse plane is as shown in Fig. 4 and 8 the accumulated dose received by the endosteal surface at the lateral medial corner at different times after injection or start of $^{90}Sr$ feeding, has been calculated and is illustrated in Fig. 10. In the case of the fed animals the accumulated dose received by the endosteal surface over the period of the experiment is about 22,000 rads, in the case of the injected animals it is less, about 13,000 rads.

On the other hand, in the injected rabbits, the shafts were in general only about $\frac{3}{4}$ the diameter of the controls indicating at some stage inhibition of transverse growth, whereas in the rabbits fed $^{90}Sr$ daily, the bones were of normal thickness. This is probably related to the dose-rate pattern which is very different in the two cases. In the case of the fed animals the surfaces receive an initial dose-rate of only 0·13 rads/hour which increases gradually to about 4 rads/hour at 6 months. In the case of the injected rabbits it falls from an initial high dose-rate (it is approximately half the maximum in 30 days) as the $^{90}Sr$ deposit becomes buried by new bone. This initial high dose-rate of about 6 rads/hour to the young growing cells of the periosteal and endosteal surfaces at the age of 5–8 weeks may account for the inhibition of transverse growth in the case of the injected animals.

A further point of interest is the fact that no abnormal bone similar to that on the endosteal surfaces was seen on the periosteal surfaces of the pellet fed animals. Because of the cylindrical shape of the bone itself, the periosteal surfaces on the whole received a somewhat smaller dose than the endosteal surfaces though this is probably not sufficient to account for the differences in damage. Other differences between the two surfaces must also be important, for instance differences in oxygen supply.

Some unpublished observations of the authors indicate that the shortening of the tibiae in the injected rabbits is due mainly to the initial high dose-rate received by the sensitive growing tissues of the epiphyseal plate. It is likely also that the gross failure of resorption and resulting damage in this region is mainly due to this high dose-rate.

*Marrow*

In the injected animals there were no areas of active haemopoiesis, the marrow showed a complete gelatinous degeneration associated with profound anaemia and, with one exception, leucopaenia. In the animals fed $^{90}Sr$ there was considerable aplasia of the marrow but areas of active haemopoiesis were always found. Some small areas of mucoid degeneration were found in only one animal. There was no significant difference in the blood picture in the two groups, but the number of animals in both cases was small.

It is difficult to quantitate the marrow damage in the two series in any way. It can only be said that the damage was more severe in the case of the injected animals. The accumulated dose to the middle of the marrow in both series has about half the value of that received in the bone wall and follows the same pattern along the bone length (Fig. 9). With regard to the more severe damage in the case of the injected animals again the different dose-rate pattern may be significant. In the case of the injected animals a limited region of sensitive bone and marrow tissues just below the epiphyseal plate at the time of injection receive an initial very heavy dose-rate which then falls off with time after injection. In the case of the rabbits fed $^{90}Sr$ the dose-rate builds up gradually from zero with time after start of feeding.

### *Calculation of the Maximum Value of the Accumulated Dose from the Terminal Burden of* $^{90}Sr$

Often the only available information is the burden of $^{90}$Sr in the bone. It was considered of interest, therefore, to compare the estimate of the maximum value of the accumulated dose made from the present measurements, with that calculated using the terminal burden of $^{90}$Sr in the tibia and the non-uniformity factor, i.e. the ratio of the maximum dose-rate to the average dose-rate.

The terminal burden of $^{90}$Sr in the tibia was known from radio-chemical measurements for three of the rabbits, 902, 930 and 932. The average dose-rates in the tibia were calculated from this, assuming uniform distribution of the $^{90}$Sr and taking into account the limited dimensions of the bone, and the non-uniformity factors were taken as 6·3 and 2 for the injected and pellet-fed rabbits respectively (Owen and Vaughan, 1959). The maximum value of the accumulated dose calculated using these quantities is given by—

Time $\times$ average radiation dose-rate $\times$ non-uniformity factor. Time is the period for which the strontium remains in the bone. Using these figures a comparison of the calculated with the measured value of the maximum accumulated dose is made in Table III.

TABLE III.—*Maximum Value of Accumulated Dose*

| | Injected | Pellet Fed. | |
|---|---|---|---|
| Rabbit number | 902 | 930 | 932 |
| Calculated | 40,824 | 40,320 | 27,216 |
| Measured | 68,600 | 22,600 | 26,673 |

In the above cases it can be seen that the maximum accumulated dose calculated from the $^{90}$Sr burden in the bone may differ from the measured value by a factor of 2. It is not possible to say whether this approaches an outside limit for the discrepancy since we are conscious of the scanty nature of the results particularly for the non-uniformity factors, which, furthermore, have been measured only for the tibia. In the injected animals the value was obtained from only one rabbit and in the fed animals it is an average of 2 rabbits. However for a variety of young and old rabbits non-uniformity factors measured in the tibia did not have a value greater than 7. What is important to remember though, is that the accumulated dose gives no indication of the different dose-rates to which the tissues may have been subjected throughout the period of exposure to the isotope. In particular, in the injected rabbits the maximum dose-rates immediately after injection were of the order of 7 times the terminal maximum dose-rate at 9 months after injection.

### DISCUSSION

The chief difficulty in determining the radiation dose in the present experiments occurred because of the variability of the rabbits themselves. For the same time interval rabbits received a wide range of dose-rates resulting from differences in uptake and retention of $^{90}$Sr. The estimate of the accumulated dose received by any site is thus subject to considerable uncertainty. Furthermore, as shown in a previous paper (Owen and Vaughan, 1959) the long range of the $\beta$-particle from $^{90}$Sr + $^{90}$Y results in a considerable radiation dose being received throughout all the bone and marrow and surrounding tissue following even a single injection. These factors make the interpretation of the results difficult and it is not possible from our experiments to give any precise answer to the interesting questions, what is the tissue, how large a volume must be irradiated and how great a dose must be received in order to produce a bone tumour.

There are strong indications from the damage described in these rabbits in the previous two papers, that it is the osteogenic connective tissues covering the bone surfaces (this includes the surfaces of the canals and cavities within the bone wall as well as the periosteal and endosteal surfaces) which are important from the point of view of tumour production. This tissue is, of course, widely distributed throughout the bone. There appeared, however, to be some correlation between the different patterns of damage and accumulated dose in the two cases studied. In the injected animals the maximum value of the accumulated dose and the greatest damage were limited to a small region of the bone, in the fed animals they were widespread over a large length of the bone examined.

Until more is known of the mechanism of the production of tumours it will be impossible to determine exactly the site of tumour origin, if indeed the latter term has any real meaning. Furthermore, it is also too simple an approach to a complex problem to try to relate damage to accumulated dose only, since it is well known that dose-rate is also important; this is confirmed in the present experiments as is discussed below.

It appeared that some of the most striking differences in damage could be attributed to the differences in dose-rate to the relevant tissues. The rabbits given a single injection received an initial heavy dose-rate to the tissues just below the epiphyseal plate at the time of injection, which then falls off with time. In the rabbits fed $^{90}$Sr daily, the dose-rate builds up gradually from zero to a maximum with time. The damage produced in both cases is severe but different. In the case of the single injection there is gross interference with cartilage resorption together with abnormal bone formation in association with the limited region of maximum dose-rate. Much of this bone and cartilage is dead but on the periphery there is found abnormal bone which is living. In the case of the fed animals where the dose-rate is lower and more uniform throughout the bone there is no obvious failure of the normal process of resorption and no excess of dead bone. Abnormal bone formation is present but is more widespread, occurring along the length of the endosteal surfaces. Tumours, however, were the end result in both cases. It is well known that tissue damage is dependent on dose-rate as well as total dose and our experiments are in agreement with this.

### SUMMARY

1. The dose-rate pattern in bone and marrow of the upper half of the tibia has been measured in rabbits given a single injection or started daily feeding of $^{90}$Sr at the age of 5–8 weeks.

2. The patterns of accumulated dose and of dose-rate have been compared with the patterns of radiation damage found in the two cases.

3. In the animals given an injection of $^{90}$Sr the maximum value of the accumulated dose and the greatest damage were limited to a small region of the bone at the level of the epiphyseal plate at the time of injection. In the animals fed $^{90}$Sr the maximum value of the accumulated dose was received over a large length of the bone examined and the greatest damage was found widespread along the endosteal surfaces. Both regions of greatest damage appeared to correspond to the sites of tumour origin.

4. Some features of the damage could be related to the different patterns of dose-rate. In the injected animals the dose-rate fell from an initial high value, in the fed animals the dose-rate increased gradually from a low value. In the case of the injected animals the initial high dose-rate caused gross failure of cartilage resorption and inhibition of longitudinal and transverse growth. No obvious failure of resorption or stunting of growth were seen in the fed animals.

It is a pleasure to acknowledge the technical assistance of Miss Jeanne Gardner.

### REFERENCES

BRUES, A. M.—(1949) *J. clin. Invest.*, **28**, 1286.
DOWNIE, E., MACPHERSON, S., RAMSDEN, E., SISSONS, H. AND VAUGHAN, J.—(1959) *Brit. J. Cancer*, **13**, 408.
FINKEL, M. P.—(1956) *Radiology*, **67**, 665.
*Idem*, LISCO, H. AND BRUES, A. M.—(1955) Quarterly Report of Biological and Medical Research Division, Argonne National Laboratory, ANL-5378, 106.
OWEN, M., JOWSEY, J. AND VAUGHAN, J.—(1955) *J. Bone Jt. Surg.*, **37 B**, 324.
*Idem*, SISSONS, H. AND VAUGHAN, J.—(1957) *Brit. J. Cancer*, **11**, 229.
*Idem*, AND VAUGHAN, J.—(1959) *Brit. J. Radiology*, in press.
TUTT, M., KIDMAN, B., RAYNER, B. AND VAUGHAN, J.—(1952) *Brit. J. exp. Path.*, **33**, 207.